# A meta-analysis on the diagnostic utility of ultrasound in pediatric distal forearm fractures

**DOI:** 10.1007/s10140-024-02208-2

**Published:** 2024-02-05

**Authors:** Amir Hassankhani, Melika Amoukhteh, Payam Jannatdoust, Parya Valizadeh, Delaram J. Ghadimi, Pauravi S. Vasavada, Jennifer H. Johnston, Ali Gholamrezanezhad

**Affiliations:** 1 Department of Radiology, Keck School of Medicine, University of Southern California (USC), 1441 Eastlake Ave Ste 2315, Los Angeles, CA 90089 USA; 2 Department of Radiology, Mayo Clinic, Rochester, MN USA; 3 School of Medicine, Tehran University of Medical Sciences, Tehran, Iran; 4 School of Medicine, Shahid Beheshti University of Medical Sciences, Tehran, Iran; 5 Department of Radiology, University Hospitals Case Medical Center, Case Western Reserve University School of Medicine, Cleveland, OH USA; 6 Department of Diagnostic and Interventional Imaging, McGovern Medical School, University of Texas Health Science Center at Houston, Houston, TX USA

**Keywords:** Pediatrics, Ultrasonography, Forearm, Radius fractures, Ulna fractures

## Abstract

Pediatric distal forearm fractures, comprising 30% of musculoskeletal injuries in children, are conventionally diagnosed using radiography. Ultrasound has emerged as a safer diagnostic tool, eliminating ionizing radiation, enabling bedside examinations with real-time imaging, and proving effective in non-hospital settings. The objective of this study is to evaluate the diagnostic efficacy of ultrasound for detecting distal forearm fractures in the pediatric population. A systematic review and meta-analysis were conducted through a comprehensive literature search in PubMed, Scopus, Web of Science, and Embase databases until October 1, 2023, following established guidelines. Eligible studies, reporting diagnostic accuracy measures of ultrasound in pediatric patients with distal forearm fractures, were included. Relevant data elements were extracted, and data analysis was performed. The analysis included 14 studies with 1377 patients, revealing pooled sensitivity and specificity of 94.5 (95% CI 92.7–95.9) and 93.5 (95% CI 89.6–96.0), respectively. Considering pre-test probabilities of 25%, 50%, and 75% for pediatric distal forearm fractures, positive post-test probabilities were 83%, 44%, and 98%, while negative post-test probabilities were 2%, 6%, and 15%, respectively. The bivariate model indicated significantly higher diagnostic accuracy in the subgroup with trained ultrasound performers vs. untrained performers (*p* = 0.03). Furthermore, diagnostic accuracy was significantly higher in the subgroup examining radius fractures vs. ulna fractures (*p* < 0.001), while no significant differences were observed between 4-view and 6-view ultrasound subgroups or between radiologist ultrasound interpreters and non-radiologist interpreters. This study highlighted ultrasound’s reliability in detecting pediatric distal forearm fractures, emphasizing the crucial role of expertise in precisely confirming fractures through ultrasound examinations.

**Supplementary Information:**

The online version contains supplementary material available at 10.1007/s10140-024-02208-2.

## Introduction

Distal forearm fractures in the pediatric population represent a significant share of musculoskeletal injuries, comprising around 30% of all fractures in children [1, 2]. The conventional diagnostic strategy predominantly involves radiography. However, this method presents notable limitations, including the potential for ionizing radiation exposure, the need for patient mobility during imaging, and challenges in identifying subtle fractures within the pediatric skeletal structure [3–5].

In recent years, ultrasound has emerged as a promising diagnostic tool for pediatric distal forearm fractures. Differing from conventional radiography, ultrasound offers a safer option for the pediatric population by eliminating ionizing radiation, which poses potential adverse effects [3, 6]. Its bedside applicability not only eliminates the need for patient transportation but also enables real-time imaging, potentially expediting the diagnostic process [7, 8]. Additionally, ultrasound presents advantages such as lower cost and higher availability [4, 9].

Recent studies have investigated the application of ultrasound in diagnosing pediatric forearm fractures, highlighting its capacity for dynamic, multi-planar visualization of both radial and ulnar cortices [10–13]. This dynamic capability effectively addresses challenges related to overlapping bone structures and provides advantages in identifying cortical disruptions and subperiosteal hematomas [7]. Moreover, the mobility of ultrasound facilitates bedside examinations, proving valuable in non-hospital settings and situations involving multiple-trauma patients [7, 8]. However, despite promising findings, the incorporation of diagnostic ultrasound into the clinical management of pediatric distal forearm fractures remains limited [4].

This study aims to conduct a meta-analysis on the diagnostic effectiveness of ultrasound in pediatric distal forearm fractures. Through a systematic review of the current literature, our objective is to offer insights into the viability of ultrasound as a diagnostic tool in the pediatric emergency setting.

## Methods

This systematic review follows the guidelines outlined in the preferred reporting items for systematic reviews and meta-analyses (PRISMA) statement [14]. On October 1, 2023, a comprehensive literature search was conducted across four major databases: PubMed, Scopus, Web of Science, and Embase. Specific search terms were devised for each database, incorporating (“radius” OR “radial” OR “ulna” OR “ulnar” OR “forearm”) AND (“fracture*” OR “displace*”) AND (“sonograph*” OR “ultrasonograph*” OR “ultrasound” OR “POCUS”) AND (“pediatric*” OR “paediatric*” OR “child*” OR “neonat*” OR “infant*” OR “toddler*” OR “preschool” OR “pre-school” OR “juvenile” OR “young adult*”). Additionally, a thorough manual examination of references within the included studies ensured no relevant papers were inadvertently overlooked. The assessment process involved a detailed review of each article’s title, abstract, and/or full text. Two co-authors independently conducted this review, resolving uncertainties or ambiguities through consultation with a senior co-author. The AutoLit platform, developed by Nested Knowledge in St. Paul, Minnesota, USA, facilitated deduplication, screening, and data extraction.

All studies pertinent to the topic of interest, presenting at least one of the following diagnostic accuracy measures in pediatric patients (under 21 years old), were considered for inclusion: sensitivity, specificity, positive predictive value (PPV), negative predictive value (NPV), likelihood ratio (LR), diagnostic odds ratio (DOR), and area under the receiver operating characteristic curve (AUC). No constraints were imposed on publication date, country of origin, patient characteristics, reference standard type, or study design. Non-English literature, case reports, case series with fewer than five eligible patients, conference abstracts, editorial comments, and review articles were excluded from the study.

The quality assessment of diagnostic accuracy studies-2 (QUADAS-2) tool was applied to evaluate the quality of included studies [15]. The four primary domains of the QUADAS-2 tool, including patient selection, index test, reference standard, and flow and timing, underwent independent assessment for potential bias and concerns regarding applicability. Evaluations for each domain were based on specific criteria outlined in the tool, such as the representativeness of the study population, blinding of test results, and completeness of outcome data. Ratings of “low,” “high,” or “unclear” were assigned to each domain to determine the overall rigor and reliability of the evidence synthesis.

### Statistical analysis

The primary analytical approach utilized a random effects diagnostic test accuracy (DTA) model, specifically the bivariate model developed by Reitsma et al. [16]. Summary receiver operating characteristic (SROC) curves were generated using this bivariate meta-analysis data. For visualization purposes, study-specific estimates were relatively weighted in SROC plots based on the weights within the random effects univariate DOR model. AUC and its confidence interval (CI) for each subgroup were calculated using a 2000 sample bootstrapping technique based on the bivariate model [17].

To assess heterogeneity, the *I*^2^ metric was employed following the approach by Holling et al. [18]. A significant level for heterogeneity was considered for *I*^2^ confidence intervals above 25%, leading to further sensitivity analyses through the DOR univariate meta-analysis to identify and re-analyze potential outliers.

The study also investigated the influence of various covariates on the reported rates using subgroup meta-analysis and meta-regression techniques. Covariates included training status and roles of the individuals involved, image acquisition methods (either four or six views), and the age of patients. Considering the variability in reporting metrics, with some studies reporting by patient number and others by the number of bones scanned, these aspects were treated as covariates for further subgroup analyses. Subgroup comparisons were made between studies reporting on a patient-wise basis vs. those reporting on a bone-wise basis. Separate subgroup analyses were also conducted for studies reporting diagnostic accuracy metrics for fractures in the radius and ulna.

Fagan plots and LR scattergrams were utilized to assess the clinical applicability of findings. Positive LRs above ten signified suitability for confirmation, whereas negative LRs below 0.1 indicated exclusion suitability. Fagan nomograms were constructed for pre-test prevalences of 25%, 50%, and 75%, based on the bivariate Reitsma model, as detailed by Zwinderman et al. [19].

Publication bias was scrutinized using an adaptation of Egger’s regression test for DTA meta-analysis, involving the analysis of funnel plot asymmetry with 2000 sample bootstrapping, as recommended by Noma et al. [20].

All statistical procedures were conducted using R (version 4.2.1, R Foundation for Statistical Computing, Vienna, Austria), utilizing packages such as “Mada,” “MVPBT” [21], “dmetatools” [17], “Metafor” [22], and “meta” [23].

## Results

### Article screening and selection process

**Fig. 1:**
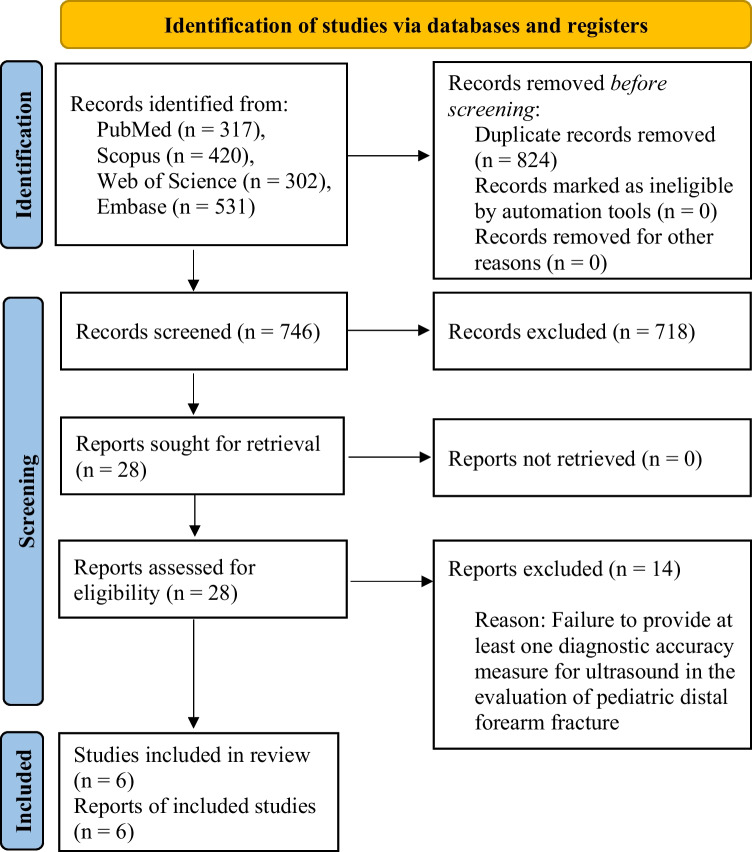
PRISMA flow diagram showing the review process. PRISMA, Preferred Reporting Items for Systematic Reviews and Meta-Analyses

A systematic literature search employing a predetermined strategy identified 1570 articles. Upon removing duplicates, 746 papers underwent screening based on title and abstract. This screening process resulted in the exclusion of 718 articles, comprising 38 review articles, 19 editorials, 15 conference papers, 49 non-English articles, and 597 articles deemed irrelevant to the topic of interest. The full text of the remaining 28 papers was meticulously reviewed. After a thorough examination, 14 articles were excluded because they failed to report at least one diagnostic accuracy measure for ultrasound in the evaluation of pediatric distal forearm fractures. Ultimately, 14 articles meeting the inclusion criteria were identified and incorporated. The screening process and eligibility criteria adhered to PRISMA guidelines, with a flow diagram presented in Fig. 1.

### Study and patient characteristics

The analysis encompassed 14 studies involving a total of 1377 patients. The predominant methodology across these studies was prospective and single center. Distal forearm fractures were diagnosed using ultrasound, with radiography as the reference standard. Diagnostic criteria for fractures on ultrasound included the identification of cortical disruptions, protrusions, deviations, or hematomas. The studies utilized a diverse range of ultrasound equipment, with a linear transducer being the most commonly employed. Additionally, a 6-view imaging protocol was predominantly implemented. The ultrasound examinations were conducted by various professionals, such as traumatology residents, pediatric emergency physicians, nurses, and radiologists, each with varying levels of training in musculoskeletal ultrasound examination. Detailed sensitivity and specificity percentages, along with additional characteristics of the included studies, can be found in Table 1.

**Table 1:** Characteristics of the included studies

Author, year	Study design	Age	Patients (F), *n*	Reference test	Fractures, *n*	Criteria for fracture on US	US device model	Probe type, frequency	4/6-view	Performer	Training	Sensitivity (%)	Specificity (%)
Herren, 2015 [13]	Prospective two center	9.5, (4 − 11) Mean, (range)	201 (69)	Radiography	R: 95 U: 15	Presence of cortical gap, cortical bulging, cortical deviation, or a positive hematoma covering the corticalis in all views	Siemens AG, RegionWest, Franz-Geuer-Str. 10, 50,823 Cologne, Germany	Linear transducer, 7.5 MHz	6-view	Traumatology resident	A brief 30-min training	R: 100 U: 93.33	R: 100 U: 100
Epema, 2019 [8]	Prospective single center	9.5, (3.6), (0 − 14) Mean, (SD), (range)	100 (50)	Radiography	64	Presence of a cortical disruption or irregularity	SonoSite Edge HFL38/Philips Sparq	Linear transducer/broadband linear array transducer, HFL38 × 13–6 MHz/12–4 MHz	6-view	EP	A brief 1-h training	95.31	86.11
Hedelin, 2017 [12]	Prospective single center	11, (3 − 16) Median, (range)	115 (62)	Radiography	77	Presence of a cortical gap, torus formation, or displacement	Fujiflm SonoSite, Inc. Edge	Linear transducer, 15–6 MHz	6-view	Physicians (various groups)	A brief 1.5-h training	97.4	84.38
Poonai, 2017 [9]	Cross-sectional	11, (3.3), (4 − 17) Mean, (SD), (range)	169 (81)	Radiography	76	N/A	Sonosite M20 Turbo	Linear transducer, 10–5 MHz	6-view	Pediatric EP	A brief 4-min training video and performed 25 satisfactory training scans prior to the study	94.74	93.55
Galletebeitia Laka, 2017 [3]	Prospective single center	9.1, (3.1), (0 − 15) Mean, (SD), (range)	115 (54)	Radiography	73	Presence of cortical irregularity or disruption	SonoSite M-turbo ultrasonograph (Fujifilm Sonosite Iberica, S.L., Madrid, Spain)	Linear transducer, 6–15 MHz	6-view	Pediatric EP	An EP with 3-year experience in POCUS, and a 1st-year pediatric resident who received training and performed 25 scans under the supervision	93.15	97.45
Ko, 2019 [10]	Prospective single center	9.9, (2 − 15) Mean, (range)	51 (26)	Radiography	34	Presence of any area demonstrating a cortical disruption or irregularity	Sonosite M-turbo machine	Linear transducer, 6–15 MHz	4-view	Sports medicine physicians and EP	Performers underwent 2 to 3 formal musculoskeletal US courses, engaged in monthly informal training sessions, and utilized US 2 to 5 times daily. Prior to the study, each investigator had completed a minimum of 10 successful scans	97.1	100
Eckert, 2012 [11]	N/A	8.8, (1 − 14) Mean, (range)	76 (37)	Radiography	R: 41 U: 11	N/A	N/A	Linear transducer, 10-MHz	6-view	N/A	N/A	R: 100 U: 81.82	R: 97.78 U: 96.92
Chaar-Alvarez, 2011 [7]	Prospective single center	10.3, (4.3), (1 − 17) Mean, (SD), (range)	101 (43)	Radiography	46	N/A	SonoSite Titan (SonoSite, Inc, Bothell, Wash)	Broadband linear transducer, L38/10-5YMHz	4-view	Pediatric EP	Board-certified PEM physician with extensive ultrasonography training, over 6 years of post-residency experience, and 135 h of advanced education in emergency ultrasonography	95.65	92.73
Ahmed, 2018 [6]	Prospective single center	7.2, (1 − 17) Mean, (range)	42 (17)	Radiography	30	Presence of a cortical gap, cortical bulging, a kink, a torus formation, or a displacement	N/A	N/A	6-view	EP	A brief 1-h training	93.3	92
Snelling, 2020 [5]	Prospective single center	9.5, (4 − 16) Mean, (range)	204 (98)	Radiography	R: 128 U: 36	Buckle fracture: an inward or outward bulge of the bone cortex without any cortical breach/other fractures: presence of a cortical breach	Fujifilm Sonosite Xporte, Bothell, Washington, USA	Linear transducer, HFL50xp/15–6 MHz	6-view	Nurses	A 2-h training course, and three supervised scans before study	R: 94.5 U: 61.1	R: 86.8 U: 95.8
Chen, 2007 [2]	Prospective single center	10, (2 − 21) Mean, (range)	68 (27)	Radiography	R: 46 U: 19	N/A	Sonosite 180 (Sonosite Inc, Bothell, Wash)	High-frequency linear transducer, 8–12 MHz	4-view	Pediatric EP	Trained in emergency US and underwent hands-on training in the ED for 1 month	R: 100 U: 89.47	R: 100 U: 100
Williamson, 2000 [24]	N/A	8, (2 − 13) Mean, (range)	26 (10)	Radiography	16	N/A	ATL 2000 machine (ATL Ultrasound, Bothell, USA)	Linear array probe, 10 MHz	6-view	Radiologists	A radiologist	100	100
Sinha, 2011 [25]	Prospective single center	12.7, (0 − 17) Mean, (range)	16	Radiography	4	Presence of a cortical break	N/A	7–10 MHz	4-view	EP, orthopedic and surgery residents	N/A	100	100
Ackermann, 2010 [26]	Prospective single center	8.1, (0–12) Mean, (range)	93 (49)	Radiography	77	Presence of a cortical gap, a kink, a torus formation, or a displacement	N/A	Linear array transducer, 7.5 MHz	6-view	N/A	N/A	94	99

*EP* emergency physician, *F* female, *N/A* no answer, *n* number, *R* radius, *SD* standard deviation, *U* ulna, *US* ultrasound

### Quality assessment

The methodological quality of the incorporated studies is detailed in Table 2 and Supplementary Fig. 1. A notable risk of bias was observed, mainly related to the diversity in ultrasound performers’ training levels. Additionally, five studies lacked explicit clarification regarding the ultrasound criteria for diagnosing forearm fractures.

**Table 2:** Methodological quality assessment of the included studies

Author, year	Q 1	Q 2	Q 3	Q 4	Q 5	Q 6	Q 7	Q 8	Q 9	Q 10	Q 11	Q 12	Q 13	Q 14	Q 15	Q 16	Q 17
Herren, 2015 [13]	Unclear	Yes	Yes	Low	Low	No	Yes	Low	Low	Yes	Yes	Low	Low	Yes	Yes	Unclear	Unclear
Epema, 2019 [8]	No	Yes	Yes	Low	Low	Yes	Yes	Low	Low	Yes	Yes	Low	Low	Yes	Yes	No	Unclear
Hedelin, 2017 [12]	Unclear	Yes	Unclear	Unclear	Low	Yes	Yes	High	Low	Yes	Yes	Low	Low	Yes	Yes	No	Low
Poonai, 2017 [9]	Yes	Yes	No	Low	Low	Yes	No	Low	Low	Yes	Yes	Low	Low	Yes	Yes	No	Unclear
Laka, 2017 [3]	No	Yes	Unclear	Low	Low	Yes	Yes	Low	Low	Yes	Yes	Low	Low	Yes	Yes	Unclear	Unclear
Ko, 2019 [10]	Unclear	Yes	Unclear	Unclear	Low	Unclear	Yes	Unclear	Low	No	No	Unclear	Low	Yes	Yes	Unclear	Unclear
Eckert, 2012 [11]	Unclear	Yes	Unclear	Unclear	Low	Yes	No	Low	Low	Yes	Yes	Low	Low	Yes	Yes	Unclear	Unclear
Chaar-Alvarez, 2011 [7]	No	Yes	No	Unclear	Low	Yes	No	Low	Low	Yes	Yes	Low	Low	Unclear	Yes	No	Unclear
Ahmed, 2018 [6]	No	Yes	No	Unclear	Low	Yes	Yes	Low	Low	No	Yes	Low	Low	Yes	Yes	Unclear	Unclear
Snelling, 2020 [5]	No	Yes	No	Unclear	Low	Yes	Yes	Low	Low	Yes	Yes	Low	Low	Unclear	Yes	No	Unclear
Chen, 2007 [2]	No	Yes	Unclear	Unclear	Low	Yes	No	Low	Low	No	Unclear	Unclear	Low	Unclear	Yes	Unclear	Unclear
Williamson, 2000 [24]	Unclear	Yes	Unclear	Unclear	Low	Unclear	No	Unclear	Low	Unclear	Unclear	Unclear	Low	Unclear	Yes	Unclear	Unclear
Sinha, 2011 [25]	No	Yes	Unclear	Unclear	Low	Yes	Unclear	Low	Low	Yes	Yes	Low	Low	Unclear	Yes	Unclear	Unclear
Ackermann, 2010 [26]	Unclear	Yes	Unclear	Unclear	Low	Unclear	Yes	Unclear	Low	Unclear	Unclear	Unclear	Low	Unclear	Yes	Unclear	Unclear

Q: Question

Q 1: Was a consecutive or random sample of patients enrolled?

Q 2: Was a case–control design avoided?

Q 3: Did the study avoid inappropriate exclusions?

Q 4: Could the selection of patients have introduced bias?

Q 5: Are there concerns that the included patients and setting do not match the review question?

Q 6: Were the index test results interpreted without knowledge of the results of the reference standard?

Q 7: If a threshold was used, was it pre-specified?

Q 8: Could the conduct or interpretation of the index test have introduced bias?

Q 9: Are there concerns that the index test, its conduct, or interpretation differ from the review question?

Q 10: Are the reference standards likely to correctly classify the target condition?

Q 11: Were the reference standard results interpreted without knowledge of the results of the index tests?

Q 12: Could the reference standard, its conduct, or its interpretation have introduced bias?

Q 13: Are there concerns that the target condition as defined by the reference standard does not match the question?

Q 14: Was there an appropriate interval between index test and reference standard?

Q 15: Did all patients receive the same reference standard?

Q 16: Were all patients included in the analysis?

Q 17: Could the patient flow have introduced bias?

### Meta-analysis

**Fig. 2:**
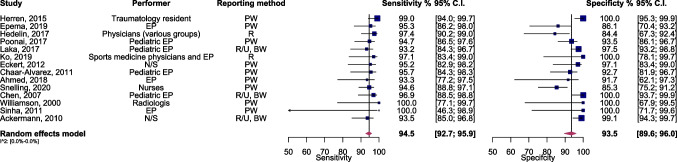
Forest plot and summary statistics of the diagnostic test accuracy (DTA) meta-analysis encompassing all included studies. CI, confidence interval; EP, emergency practitioner; N/S, not specified; PW, patient-wise; BW, bone-wise; R, radius; U, ulna

In the evaluation of pediatric distal forearm fractures across 14 studies, the pooled sensitivity and specificity were found to be 94.5 (95% CI 92.7–95.9) and 93.5 (95% CI 89.6–96.0), respectively (Fig. 2). The SROC curve demonstrated an AUC of 0.94 (95% CI 0.92–0.97) (Supplementary Fig. 2). Supplementary Fig. 3, depicting a scattergram of positive and negative likelihood ratios, suggests a high-performance level, ideal for both exclusion and confirmation purposes. According to the Fagan plot study, considering pre-test probabilities of 25%, 50%, and 75% for distal forearm fractures in children, the positive post-test probabilities were 83%, 44%, and 98%, while the negative post-test probabilities were 2%, 6%, and 15%, respectively (Supplementary Fig. 4).

### Meta-regression and subgroup analyses

Additional examination involved meta-regression and subgroup assessments, focusing on various covariates.

**Fig. 3:**
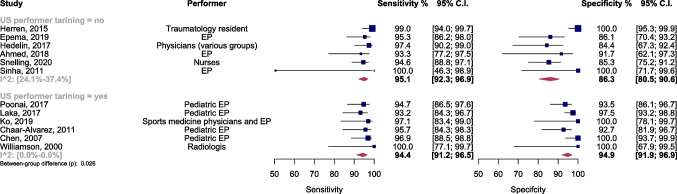
Forest plot and summary statistics of diagnostic test accuracy (DTA) subgroup meta-analysis comparing studies with trained/untrained ultrasound performers. CI, confidence interval; EP, emergency physician; US, ultrasound

**Fig. 4:**
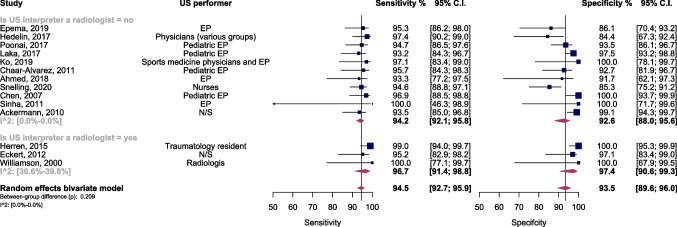
Forest plot and summary statistics of diagnostic test accuracy (DTA) subgroup meta-analysis comparing studies based on the professional background of ultrasound interpreters. CI, confidence interval; EP, emergency physician; US, ultrasound

**Fig. 5:**
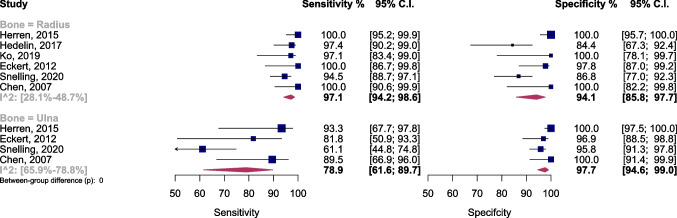
Forest plot and summary statistics of diagnostic test accuracy (DTA) subgroup meta-analysis comparing the performance of ultrasound examination to detect fractures of radius and ulna. CI, confidence interval

- Ultrasound performers’ training status
  In studies where individuals with ultrasound training conducted the examinations, the sensitivity and specificity were 94.4% (95% CI 91.2–96.5) and 94.9% (95% CI 91.9–96.9), respectively. In contrast, studies with untrained performers showed sensitivity and specificity of 95.1% (95% CI 92.3–96.9) and 86.3% (95% CI 80.5–90.6), respectively (Fig. 3). The AUC of the SROC was 0.95 (95% CI 0.93–0.98) and 0.94 (95% CI 0.86–0.97) for studies with trained and untrained ultrasound performers, respectively. Analyzing with a bivariate model indicated significantly higher diagnostic accuracy in the subgroup with trained performers (*p* = 0.03), as depicted in Supplementary Fig. 5. Furthermore, post hoc analysis revealed notably higher specificity in this subgroup (*p* < 0.01).
  Supplementary Fig. 6 illustrates a scattergram of positive and negative LRs for each subgroup.
  Moderate heterogeneity was noted in studies with untrained ultrasound performers. A sensitivity analysis identified the study by Herren et al. [13] as a significant outlier. After excluding this study, the overall diagnostic accuracy and specificity remained significantly higher in the subgroup with trained ultrasound performers (Supplementary Figs. 7 and 8).
- Ultrasound interpreter
  In studies where radiologists interpreted the ultrasound, the sensitivity and specificity were 94.5% (95% CI 92.7–95.9) and 93.5% (95% CI 89.6–96.0), respectively. In studies with ultrasound interpreters other than radiologists, the sensitivity and specificity were 94.2% (95% CI 92.1–95.8) and 92.6% (95% CI 88.0–95.6), respectively (Fig. 4). The AUC of the SROC was 0.99 (95% CI 0.93–0.99) for studies with radiologist interpreter and 0.95 (95% CI 0.92–0.97) for studies with interpreters other than radiologists. The diagnostic accuracy did not differ significantly between the two subgroups (Supplementary Fig. 9).
- Bone under examination (radius vs. ulna)
  The sensitivity and specificity of ultrasound examination for detecting radius fractures were 97.1% (95% CI 94.2–98.6) and 94.1% (95% CI 85.8–97.7), respectively. Additionally, the sensitivity and specificity of ultrasound examination for detecting ulnar fractures were 78.9% (95% CI 61.6–89.7) and 97.7% (95% CI 94.6–99.0), respectively (Fig. 5). The AUC of the SROC was 0.98 (95% CI 0.94–0.98) for studies investigating radius fractures and 0.97 (95% CI 0.79–0.99) for studies investigating ulnar fractures. Utilizing a bivariate model, it was observed that the diagnostic accuracy was significantly higher in the subgroup examining radius fractures (*p* < 0.001), as illustrated in Supplementary Fig. 10. Additionally, a post hoc analysis revealed a notably higher sensitivity in this subgroup (*p* < 0.001).
  Supplementary Fig. 11 displays a scattergram of each subgroup’s positive and negative LRs.
  Considerable heterogeneity was observed in both subgroups. A sensitivity analysis was conducted to identify potential outliers and investigate the source of this heterogeneity. This analysis identified the study by Herren et al. [13] as a significant outlier. After excluding this study, the overall diagnostic accuracy and sensitivity remained significantly higher in the subgroup of studies investigating radius fractures. However, post hoc analysis revealed that the specificity of ultrasound for the ulna subgroup was significantly higher than the other subgroup (*p* < 0.01) (Supplementary Figs. 12 and 13).
- Ultrasound views (4-view vs. 6-view)
  The sensitivity and specificity of the 4-view ultrasound examination for detecting distal forearm fractures were 95.3% (95% CI 90.5–97.7) and 94.4% (95% CI 88.1–97.5), respectively. Additionally, the sensitivity and specificity of the 6-view ultrasound examination were 94.4% (95% CI 92.2–95.9) and 92.9% (95% CI 87.9–96.0), respectively (Supplementary Fig. 14). The AUC of the SROC was 0.96 (95% CI 0.92–0.98) for the 4-view subgroup and 0.94 (95% CI 0.92–0.97) for the 6-view subgroup. The diagnostic accuracy did not differ significantly between the two subgroups (Supplementary Fig. 15).
- Reporting method (bone-wise vs. patient-wise)
  The pooled sensitivity and specificity were examined in two subgroups. In studies reporting results on a bone-wise basis, the sensitivity was 93.8% (95% CI 90.04–96.1), and the specificity was 97.0% (95% CI 93.2–98.7). In contrast, in studies reporting results on a patient-wise basis, the sensitivity was 94.7% (95% CI 92.2–96.4), and the specificity was 90.6% (95% CI 86.3–93.6) (Supplementary Fig. 16). The AUC of the SROC was 0.97 (95% CI 0.92–0.98) for the bone-wise subgroup and 0.96 (95% CI 0.91–0.97) for the patient-wise subgroup. Utilizing a bivariate model, the diagnostic accuracy was observed to be slightly higher in the bone-wise subgroup (*p* = 0.07), as demonstrated in Supplementary Fig. 17. Furthermore, a post hoc analysis indicated notably higher specificity in the bone-wise subgroup (*p* = 0.04).
- ***Age***
  In the meta-regression analysis, using a bivariate model to assess the impact of age, no significant influence of the mean age of the study samples was observed as a factor explaining heterogeneity (*p* = 0.53).

### Funnel plot analysis

In Supplementary Fig. 18, paired funnel plots illustrating publication bias and the small study effect are presented. Importantly, the application of Egger’s regression analysis with 2000 bootstrapping revealed significant asymmetry (*p* = 0.23), suggesting evidence of potential publication bias or a small study effect.

## Discussion

This systematic review and meta-analysis extend prior investigations to assess the diagnostic effectiveness of ultrasound in detecting distal forearm fractures in pediatric cases. This study reveals that ultrasonography exhibits a notable sensitivity and a high specificity in the detection of distal forearm fractures in pediatric patients. It proves to be highly accurate in positively identifying cases with fractures and effectively excluding those without. The pooled positive and negative LRs reported further underscore its reliability for both detection and exclusion purposes. These findings position ultrasonography as a valuable tool in clinically assessing distal forearm fractures in pediatrics, aiding healthcare practitioners in making well-informed decisions about patient care.

As an effective diagnostic tool for distal forearm fractures, ultrasonography leverages factors such as good image quality resulting from thin soft tissue and the short distance between the transducer and bone [6]. In contrast to radiography, ultrasonography enables the assessment of soft tissues, including muscle edema, tendons, and joint functions, providing exceptional spatial resolution and multiplanar imaging capabilities. Furthermore, ultrasonography proficiently localizes soft tissue interpositions between fracture fragments [7, 13]. Crucially, it adeptly addresses time constraints in emergency scenarios by facilitating rapid examinations. Ultrasonography eliminates the need for transporting patients to radiology suites and bypasses the subsequent wait for radiologist readings. This expedited process makes a substantial contribution to reducing the overall patient length of stay, effectively mitigating concerns associated with prolonged emergency department wait times [6]. The absence of ionizing radiation, coupled with the opportunity for families to view images during the initial physician visit, underscores the efficiency and patient-centric benefits of ultrasonography in diagnosing distal forearm fractures [7].

Research consistently indicates that ultrasound for detecting pediatric distal forearm fractures is less painful than radiographic imaging. In the study by Chaar-Alvarez et al., the pain score during ultrasound was significantly lower than during radiographic imaging [7]. This aligns with broader literature where patients consistently reported similar or lower pain scores for point-of-care ultrasound (POCUS) compared to X-ray [5]. Furthermore, in the study by Xo et al., the results suggest that pain with ultrasonography is unlikely to limit its use in this diagnostic context [9]. The minimal discomfort associated with POCUS can be attributed to its non-invasive nature, as all images can be obtained without requiring the child to move the affected extremity [5, 9]. This indicates that clinicians can reassure caregivers and children that ultrasonography is generally not a painful procedure.

Our findings indicate that the diagnostic accuracy of ultrasound did not significantly differ between the subgroups of radiologist interpreters and non-radiologist interpreters. However, a significantly higher diagnostic accuracy was observed in the subgroup with trained performers, particularly showcasing notably higher specificity. No difference was noted between trained and untrained individuals in terms of sensitivity. This suggests that ultrasound can effectively rule out distal forearm fractures in children regardless of the operator’s training level. Nevertheless, a crucial distinction emerges when confirming fractures, emphasizing the necessity of specific training. This underscores the importance of skill and expertise in accurately confirming fractures through ultrasound examination. Studies have reported that a standardized training duration of approximately 1 h is sufficient [25–27], indicating that the learning curve for bone ultrasonography is not excessively complex, at least for pediatric distal forearm fractures. The study by Galletebeitia Laka et al. demonstrates that a pediatric resident, even without prior POCUS experience, achieved diagnostic accuracy comparable to an experienced emergency department pediatrician after receiving basic training [3]. Similarly, Epema et al. demonstrated that inexperienced physicians can master POCUS of the forearm after a short training period [8]. Moreover, Chaar-Alvarez et al.’s findings confirm the accuracy of ultrasound as a diagnostic tool for nonangulated pediatric forearm fractures, even when reviewed by an experienced, blinded professional relying solely on ultrasound images for diagnosis. In contrast, unblinded bedside diagnoses by less experienced physicians were accurate but to a lesser extent. This indicates that having more ultrasound experience was more crucial and potentially less prone to misleading results than relying solely on additional bedside clinical information for making accurate diagnoses [7].

We noted a significant increase in diagnostic accuracy within the subgroup focused on examining radius fractures, with the analysis highlighting notably higher sensitivity in this category. This trend persisted even after the exclusion of an outlier study. However, upon removing this study, the specificity of ultrasound for the ulna subgroup was found to be significantly higher. Consequently, ultrasound demonstrates greater reliability in excluding radius fractures and proves more effective in confirming ulna fractures. This variability may be attributed to anatomical differences, operator experience and training, variability in patient positioning, characteristics of the study population, and differences in equipment/technology. Further investigation into these factors is necessary to gain a deeper understanding of the nuanced reasons behind the observed differences in diagnostic accuracy for fractures in distinct forearm bones in pediatric cases.

Although the current study’s findings highlighted the reliability of ultrasonography in detecting pediatric distal forearm fractures, it is crucial to acknowledge that this imaging modality may not be universally suitable, especially in cases involving patients with obesity or other factors affecting image quality. Consequently, clinical judgment should guide the choice of the most appropriate imaging modality for each individual patient. Additionally, it is worth noting that ultrasound operators might have been influenced by visible deformities or physical signs during image interpretation, potentially leading to an overestimation of ultrasound accuracy.

While this systematic review centered on the diagnostic utility of ultrasound in pediatric distal forearm fractures, questions about its role post-diagnosis—particularly its potential to inform surgical decisions and support follow-up assessments—remain unexplored. Understanding these aspects is crucial for advancing clinical practice and encourages further investigation into the broader utility of ultrasound in managing pediatric distal forearm fractures.

## Conclusions

This study demonstrated the reliability of ultrasonography as an imaging modality for detecting distal forearm fractures in pediatric patients, exhibiting high sensitivity and specificity. Notably, trained performers displayed significantly higher diagnostic accuracy, particularly in terms of enhanced specificity, underscoring the importance of expertise in accurately confirming fractures through ultrasound examination. Further research should specifically address the observed differences in diagnostic accuracy of ultrasound between fractures in the radius and ulna.

### Supplementary Information

Below is the link to the electronic supplementary material.

Supplementary file1 (DOCX 10174 KB)

## Data Availability

The datasets analyzed during the current study are available from the corresponding author on reasonable request.
