# Supplementary material for: A meta-analysis on the diagnostic utility of ultrasound in pediatric distal forearm fractures
Source: Emerg Radiol. 2024 Feb 5;31(2):213–28. doi: 10.1007/s10140-024-02208-2 (PMC10994871; doi:10.1007/s10140-024-02208-2)
Supplement: Supplementary file 1 — Supplementary file1 (DOCX 10174 KB) [file 10140_2024_2208_MOESM1_ESM.docx]

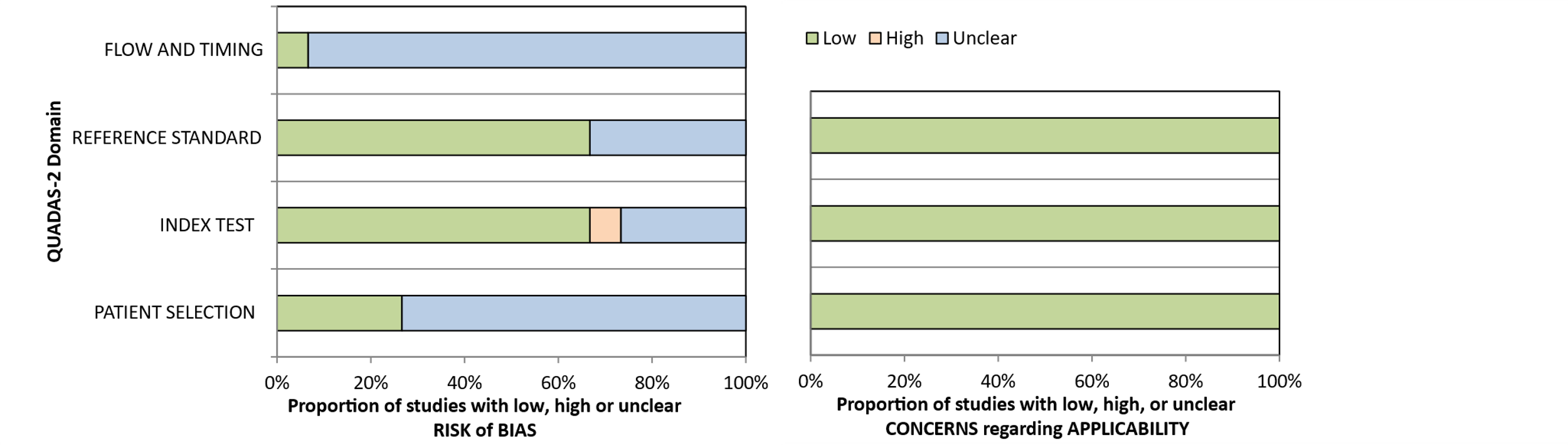
**Supplementary Fig. 1**. Overview of bias risk assessment results


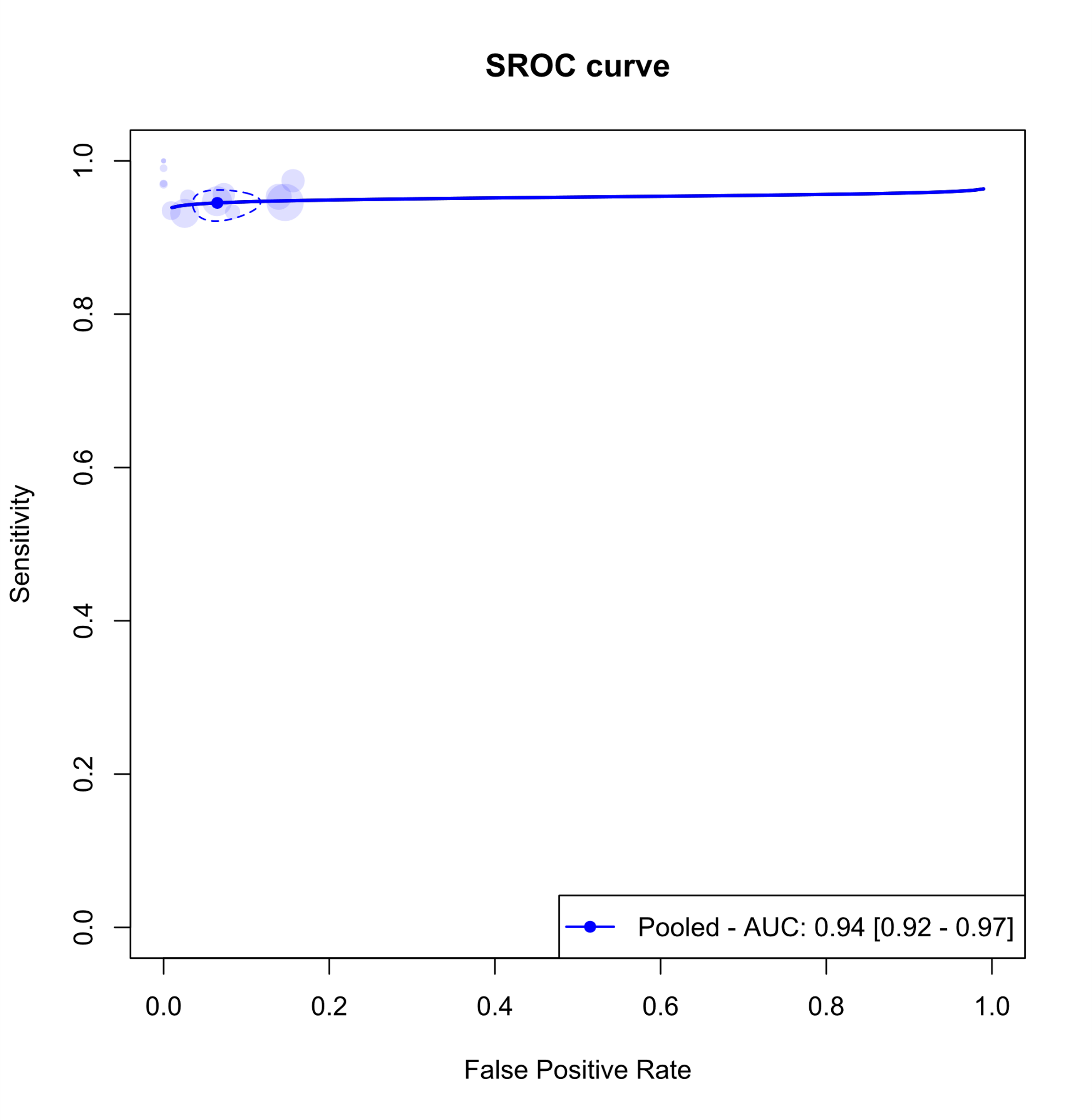
**Supplementary Fig. 2**. Summary receiver operating characteristic curve (SROC) for the diagnostic test accuracy (DTA) meta-analysis across all included studies. AUC - Area Under the Curve


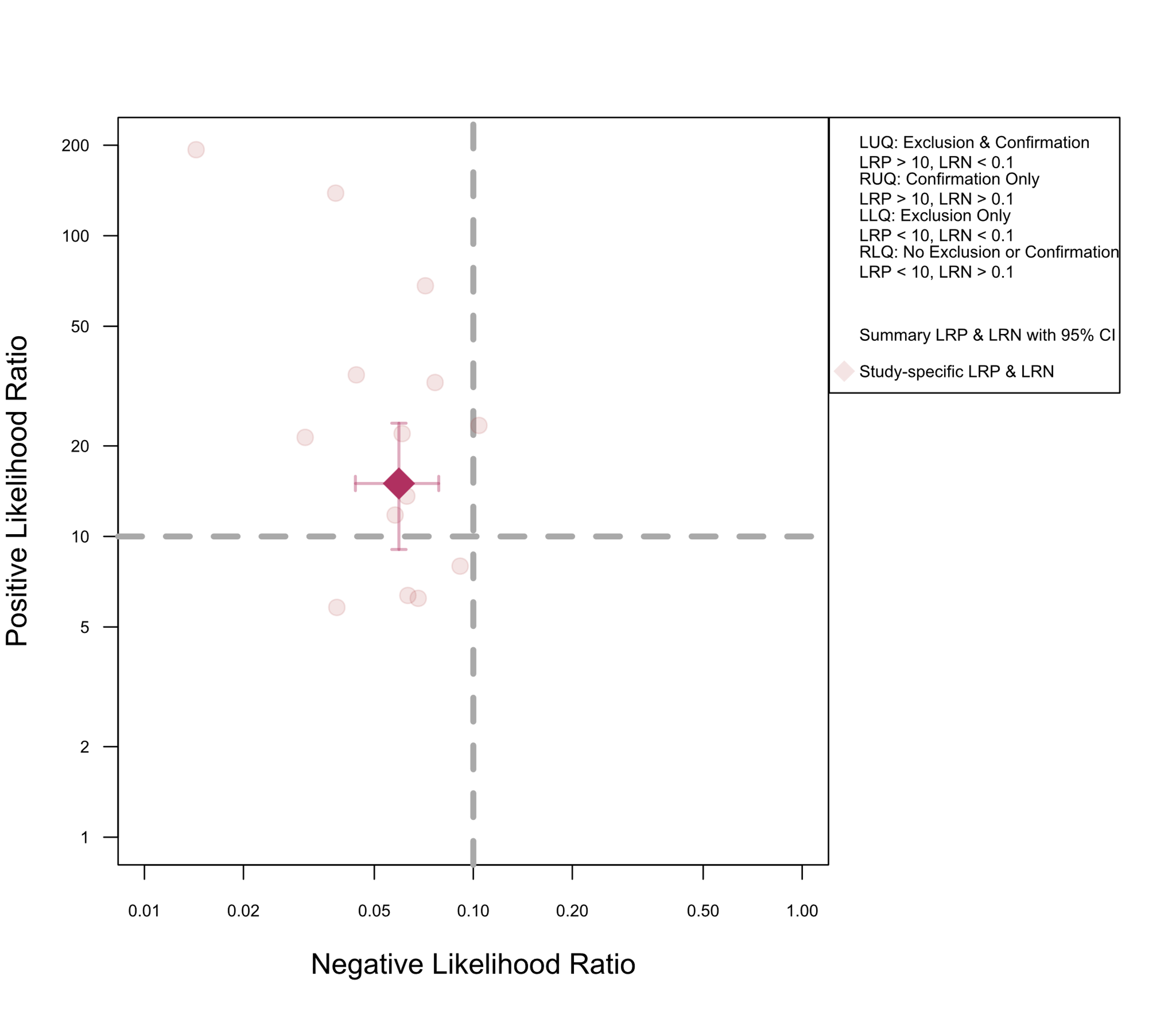
 **Supplementary** **Fig. 3**. Likelihood ratio scattergram of included studies. LLQ - Left Lower Quadrant. LRN - Likelihood Ratio, Negative. LRP - Likelihood Ratio, Positive. LUQ - Left Upper Quadrant. RLQ - Right Lower Quadrant. RUQ - Right Upper Quadrant


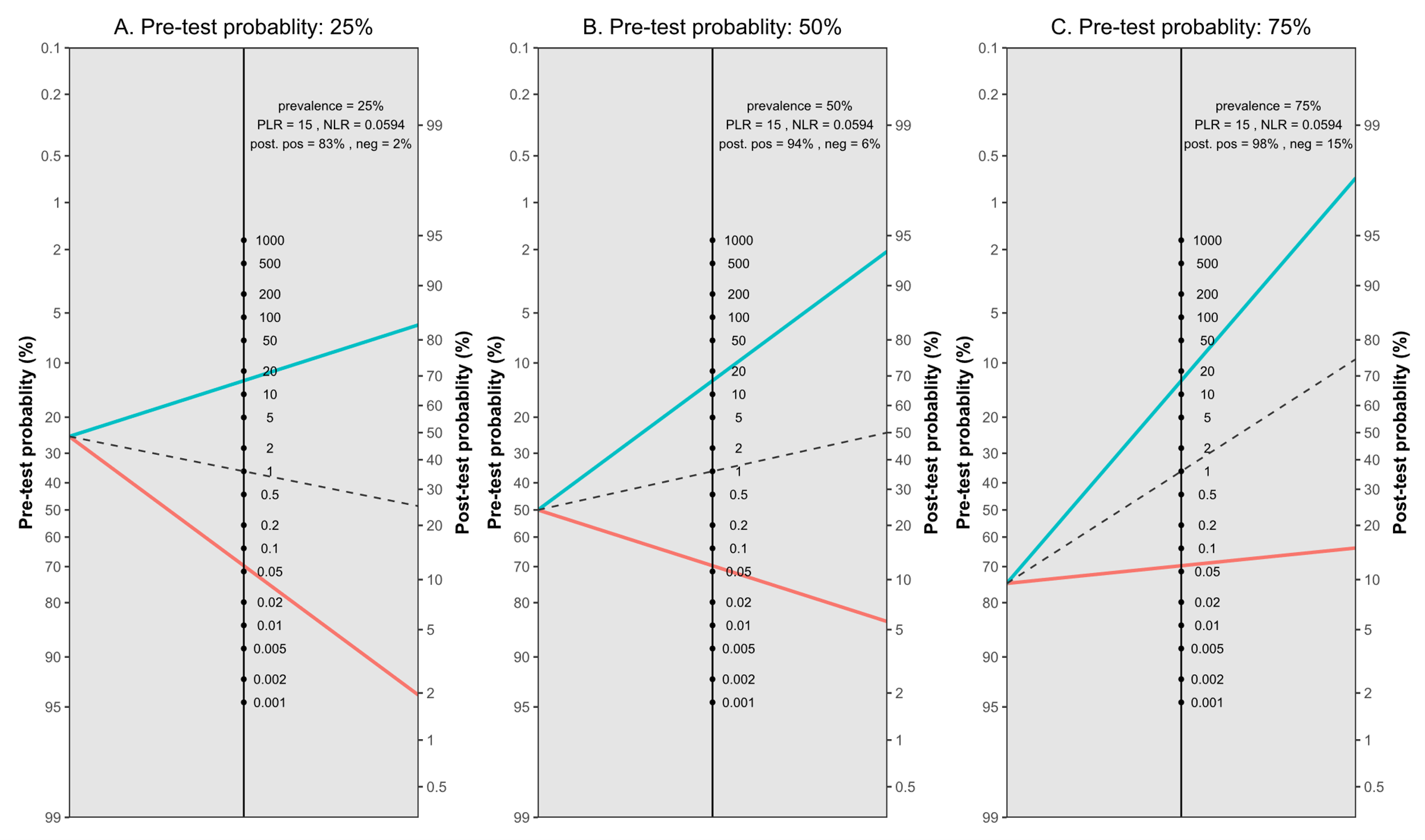


**Supplementary Fig. 4**. Fagan plot analysis utilizing summary positive and negative likelihood ratio results from the meta-analysis of all included studies, with hypothetical pre-test probabilities of 25%, 50%, 75%. PLR - Positive Likelihood Ratio, NLR - Negative Likelihood Ratio, Neg - Negative, Pos - Positive


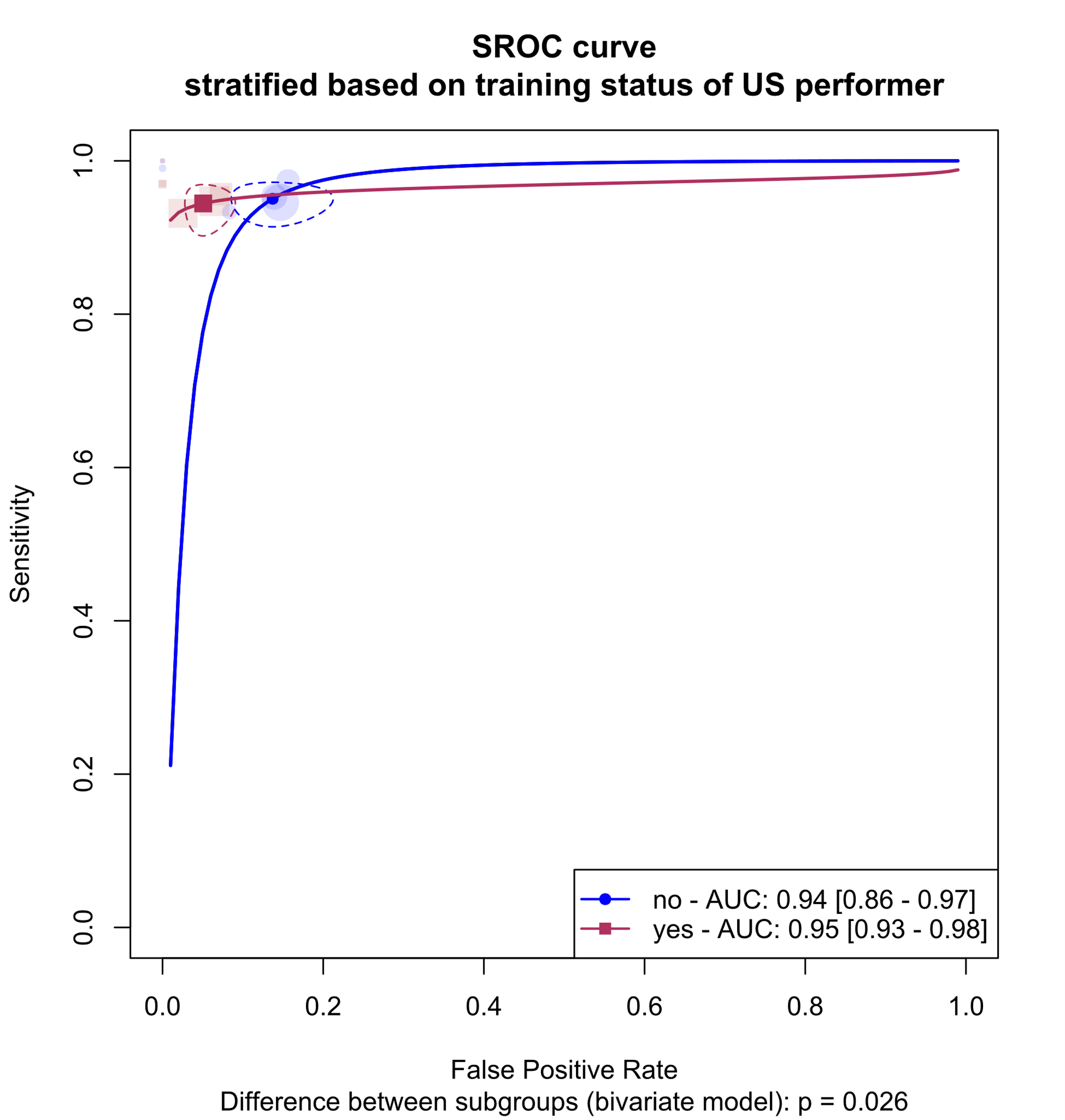


**Supplementary** **Fig. 5**. Summary receiver operating characteristic curve (SROC) for the diagnostic test accuracy (DTA) subgroup meta-analysis comparing studies with trained/untrained ultrasound performers. AUC - Area Under the Curve


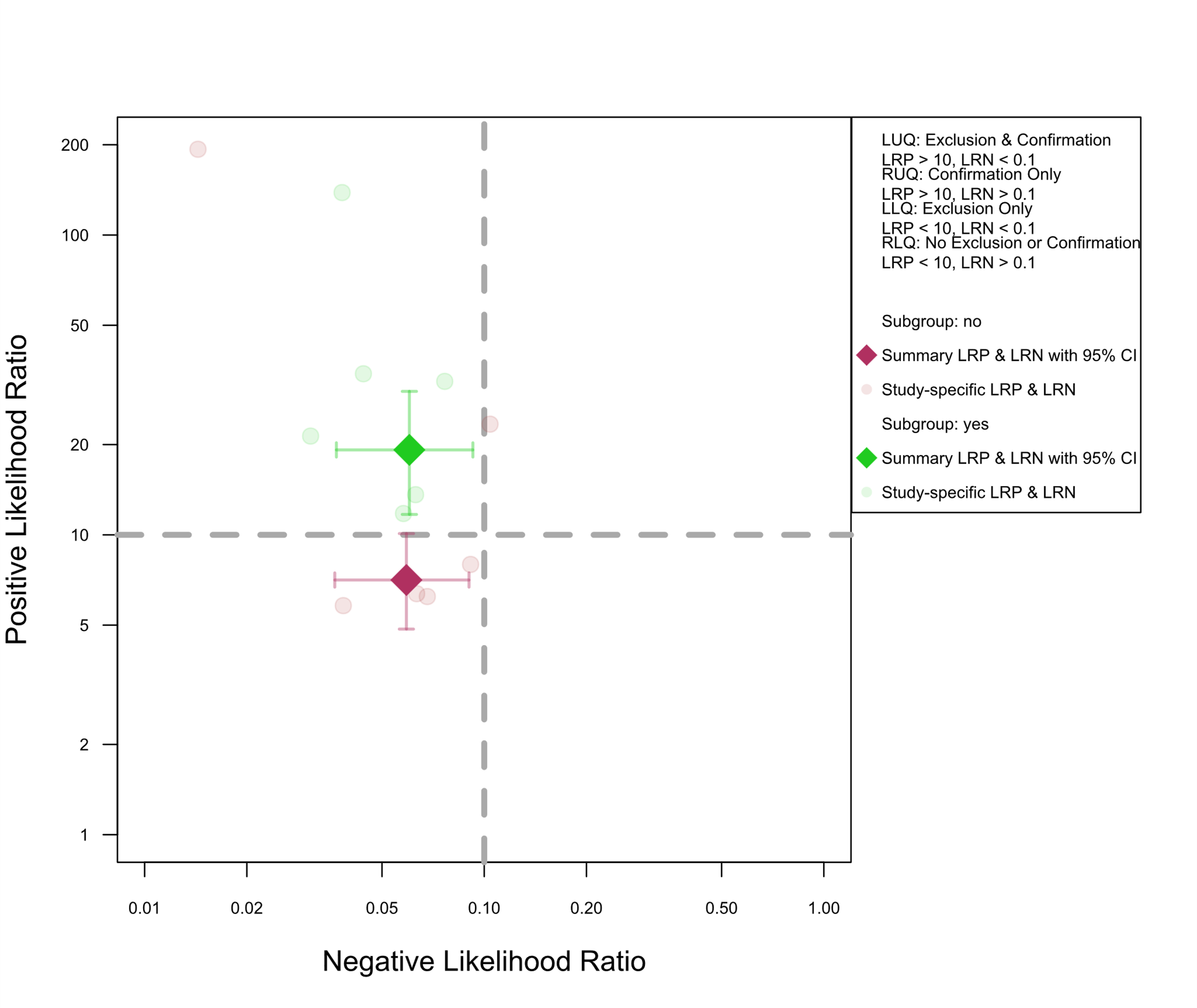


**Supplementary Fig. 6.** Likelihood ratio scattergram of included studies comparing studies with trained and untrained performers. LLQ - Left Lower Quadrant. LRN - Likelihood Ratio, Negative. LRP - Likelihood Ratio, Positive. LUQ - Left Upper Quadrant. RLQ - Right Lower Quadrant. RUQ - Right Upper Quadrant


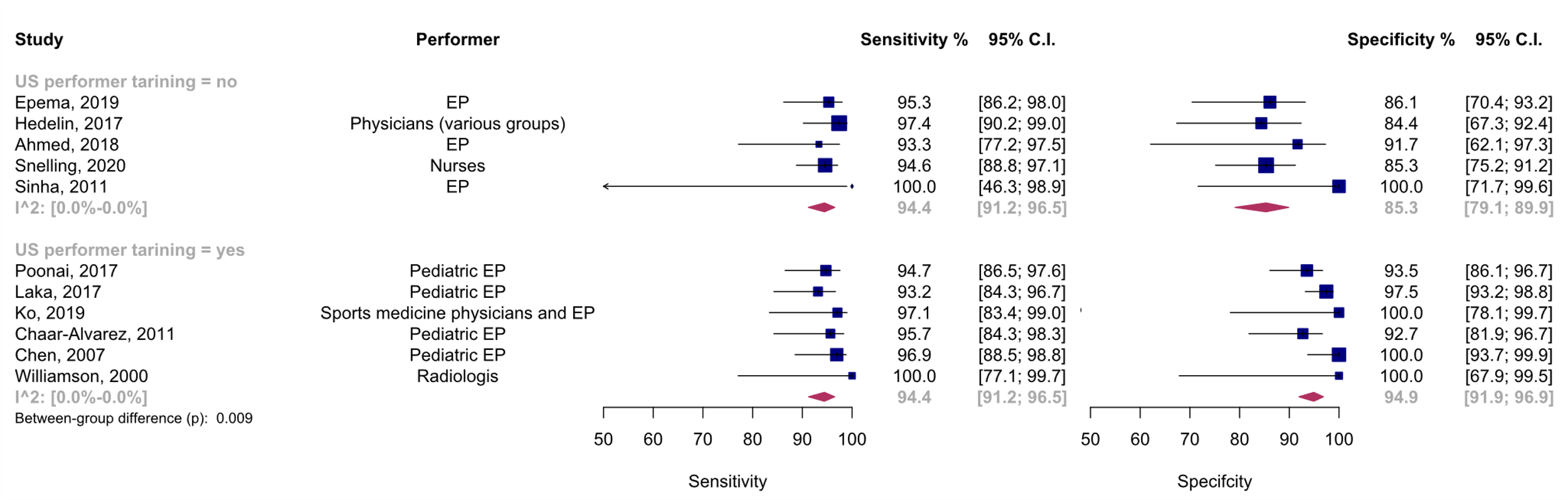
 **Supplementary Fig. 7**. Forest plot and summary statistics of diagnostic test accuracy (DTA) subgroup meta-analysis comparing studies with trained/untrained ultrasound performers after excluding one outlier study. Abbreviations: CI - Confidence Interval, EP - Emergency Physician, US - Ultrasound


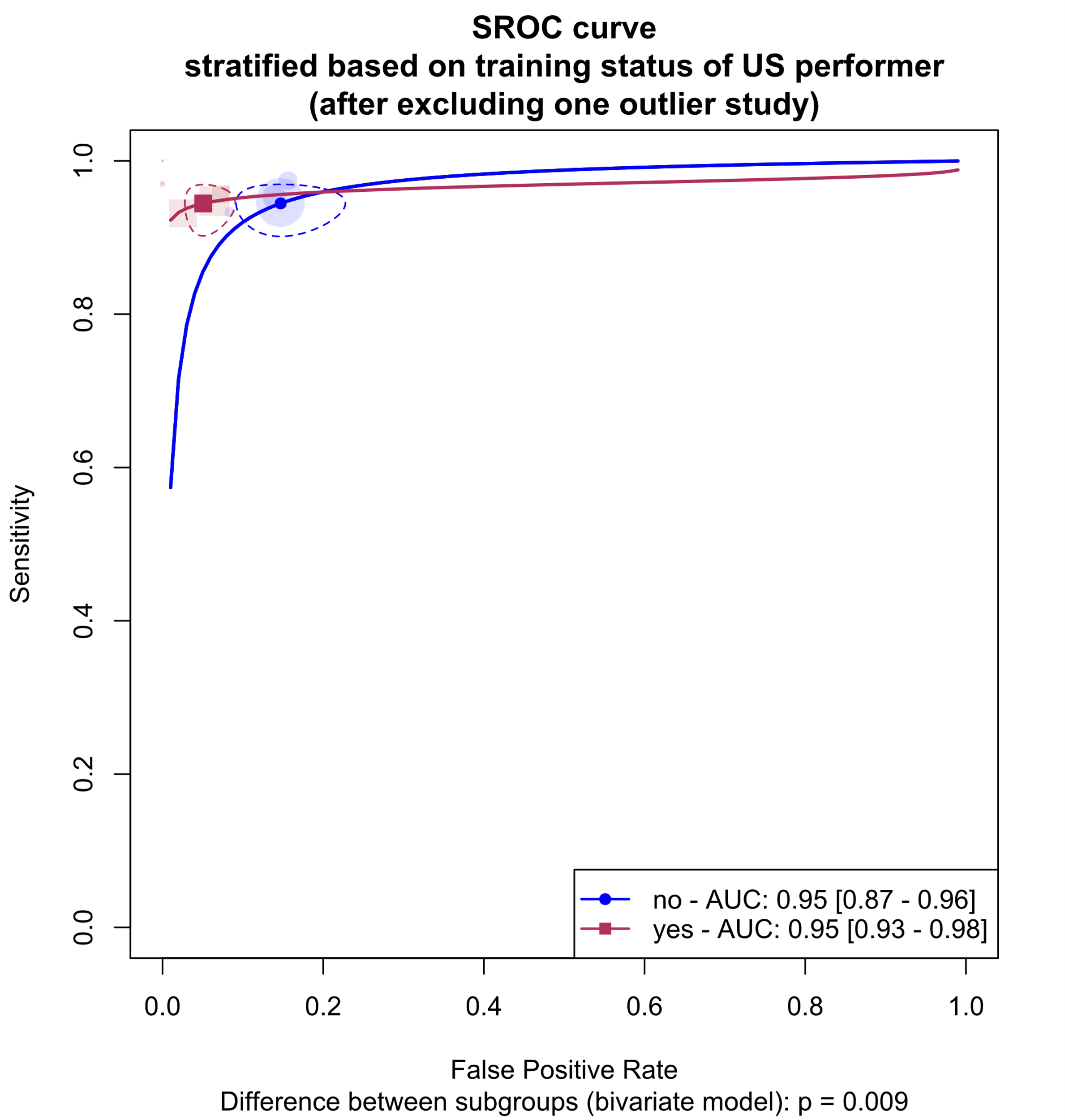


**Supplementary Fig. 8**. Summary receiver operating characteristic curve (SROC) of diagnostic test accuracy (DTA) subgroup meta-analysis comparing studies with trained/untrained ultrasound performers after excluding one outlier study. AUC - Area Under the Curve


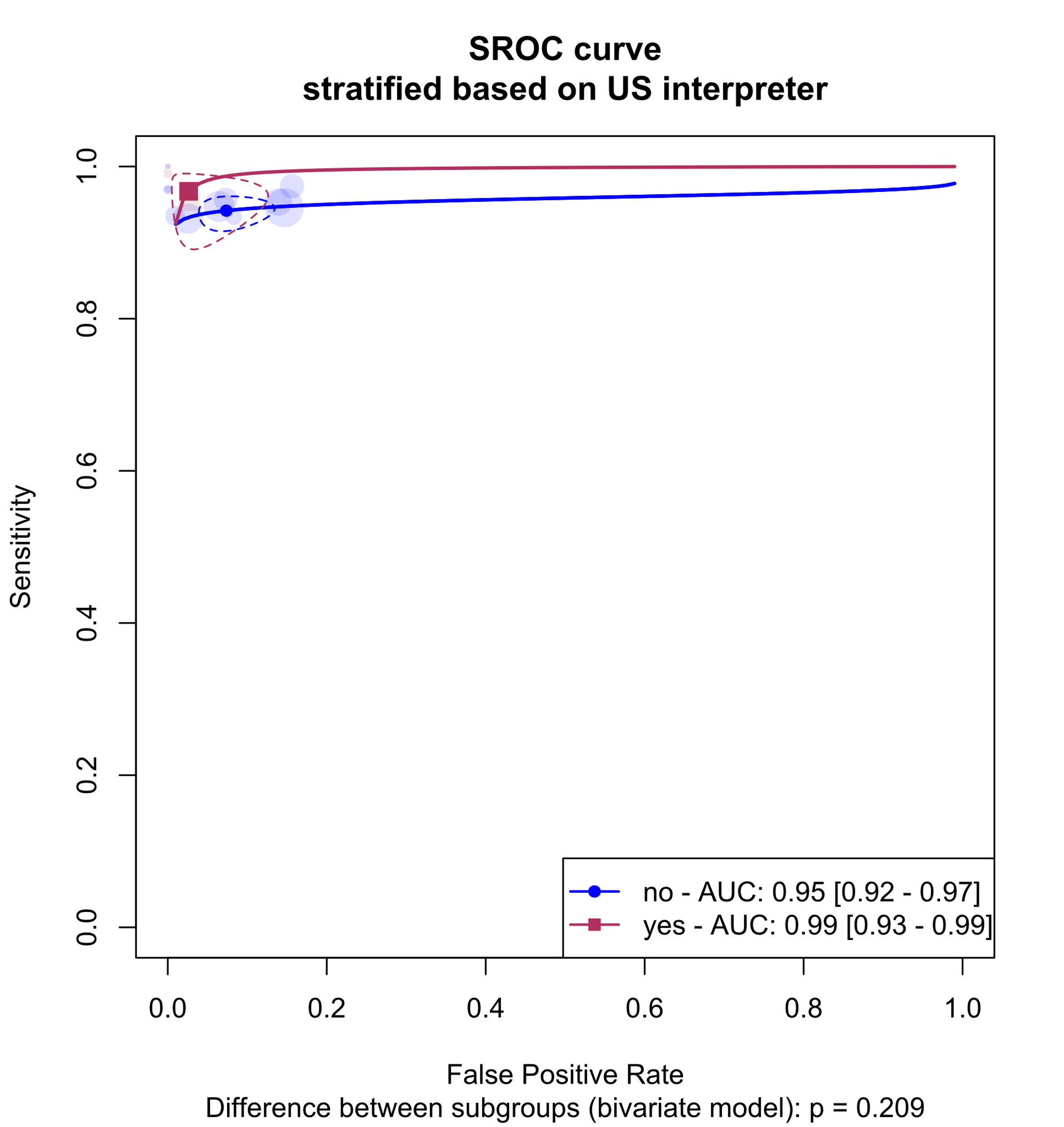


**Supplementary Fig. 9**. Summary receiver operating characteristic curve (SROC) for the diagnostic test accuracy (DTA) subgroup meta-analysis comparing studies with radiologists/other physicians as ultrasound interpreters. AUC - Area Under the Curve


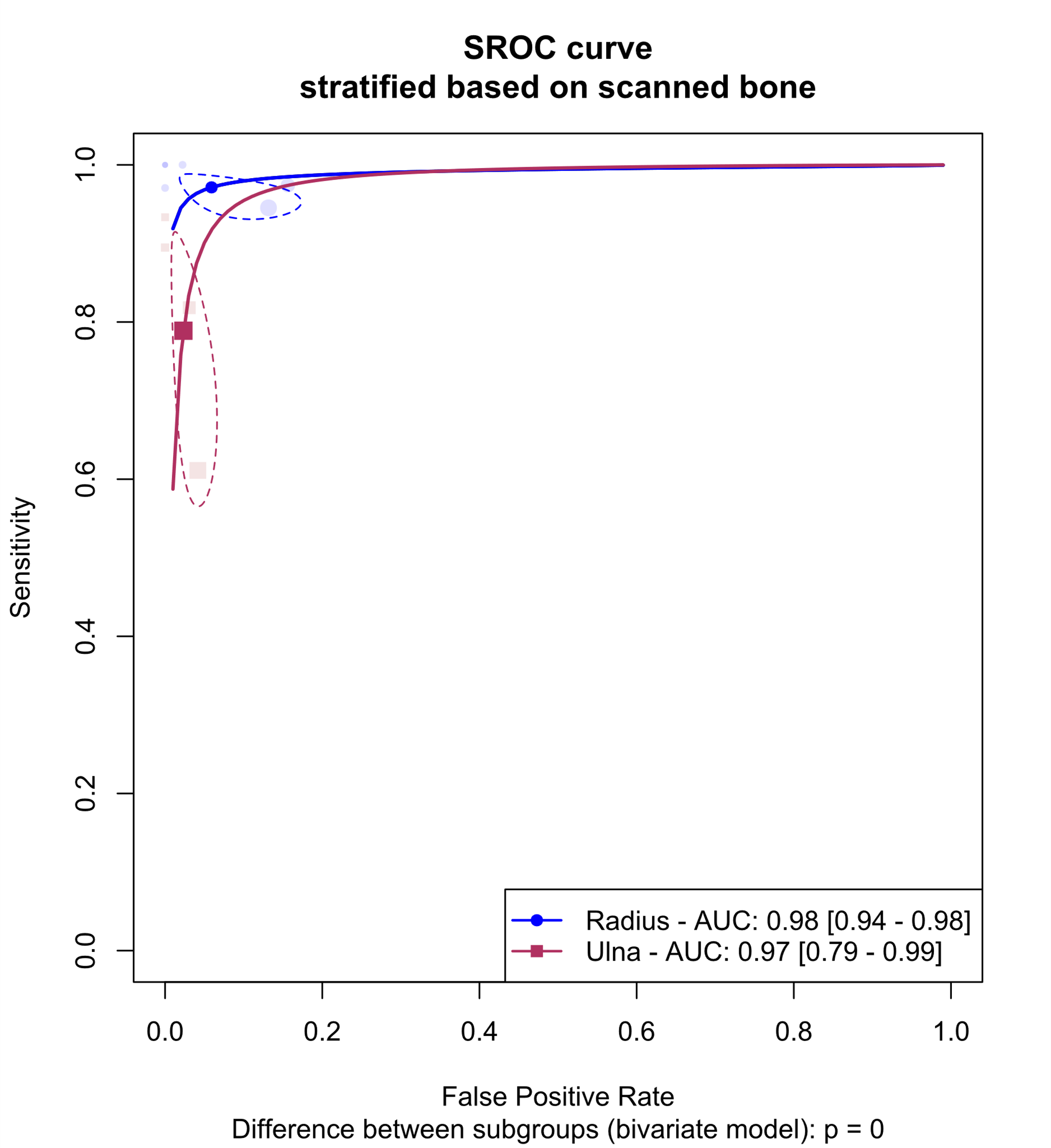


**Supplementary Fig. 10**. Summary receiver operating characteristic curve (SROC) of diagnostic test accuracy (DTA) subgroup meta-analysis comparing the performance of ultrasound in detecting the fractures of radius and ulna. AUC: Area Under the Curve


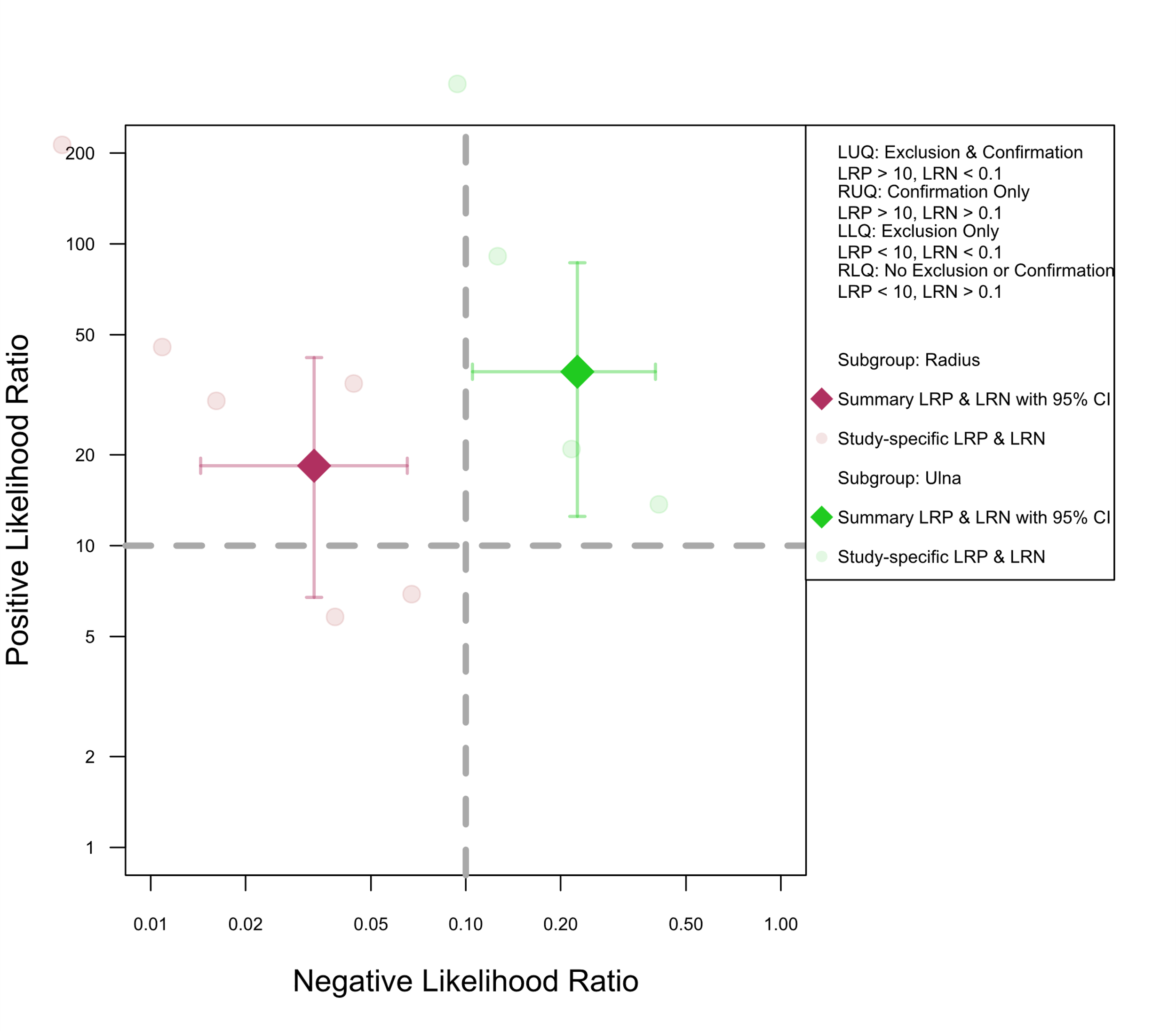


**Supplementary Fig. 11.** Likelihood ratio scattergram of included studies comparing the performance of ultrasound in detecting the fractures of radius and ulna. LLQ - Left Lower Quadrant. LRN - Likelihood Ratio, Negative. LRP - Likelihood Ratio, Positive. LUQ - Left Upper Quadrant. RLQ - Right Lower Quadrant. RUQ - Right Upper Quadrant


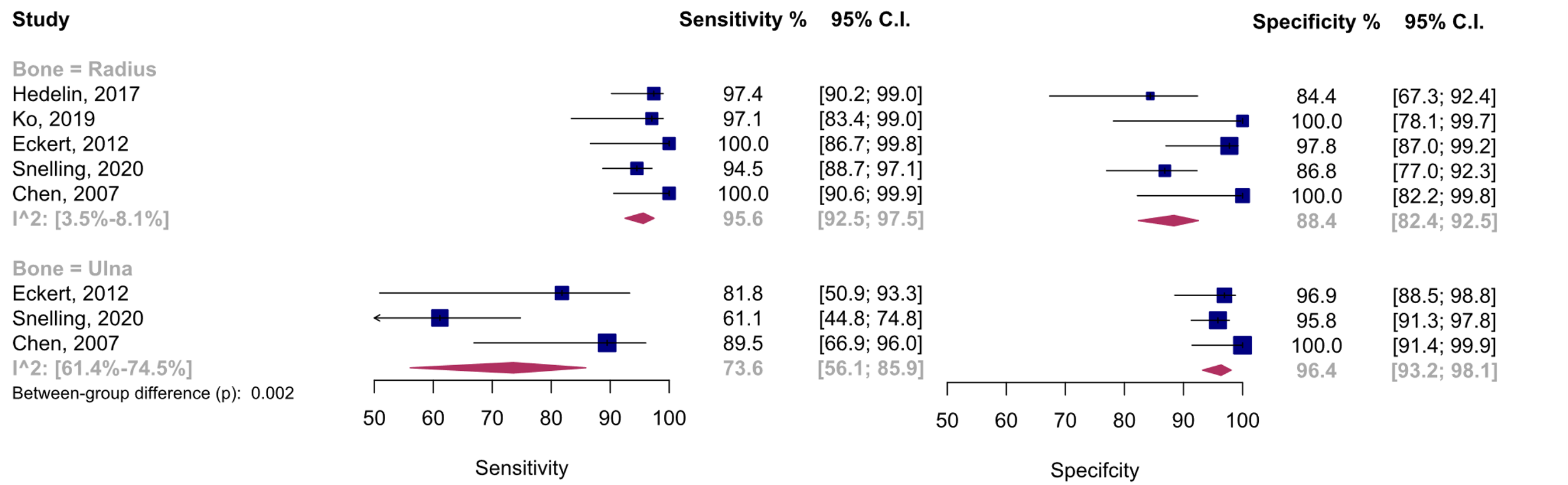


**Supplementary Fig. 12**. Forest plot and summary statistics of diagnostic test accuracy (DTA) subgroup meta-analysis comparing the performance of ultrasound to detect fractures of radius and ulna after excluding one outlier study. CI - Confidence Interval, EP - Emergency Physician


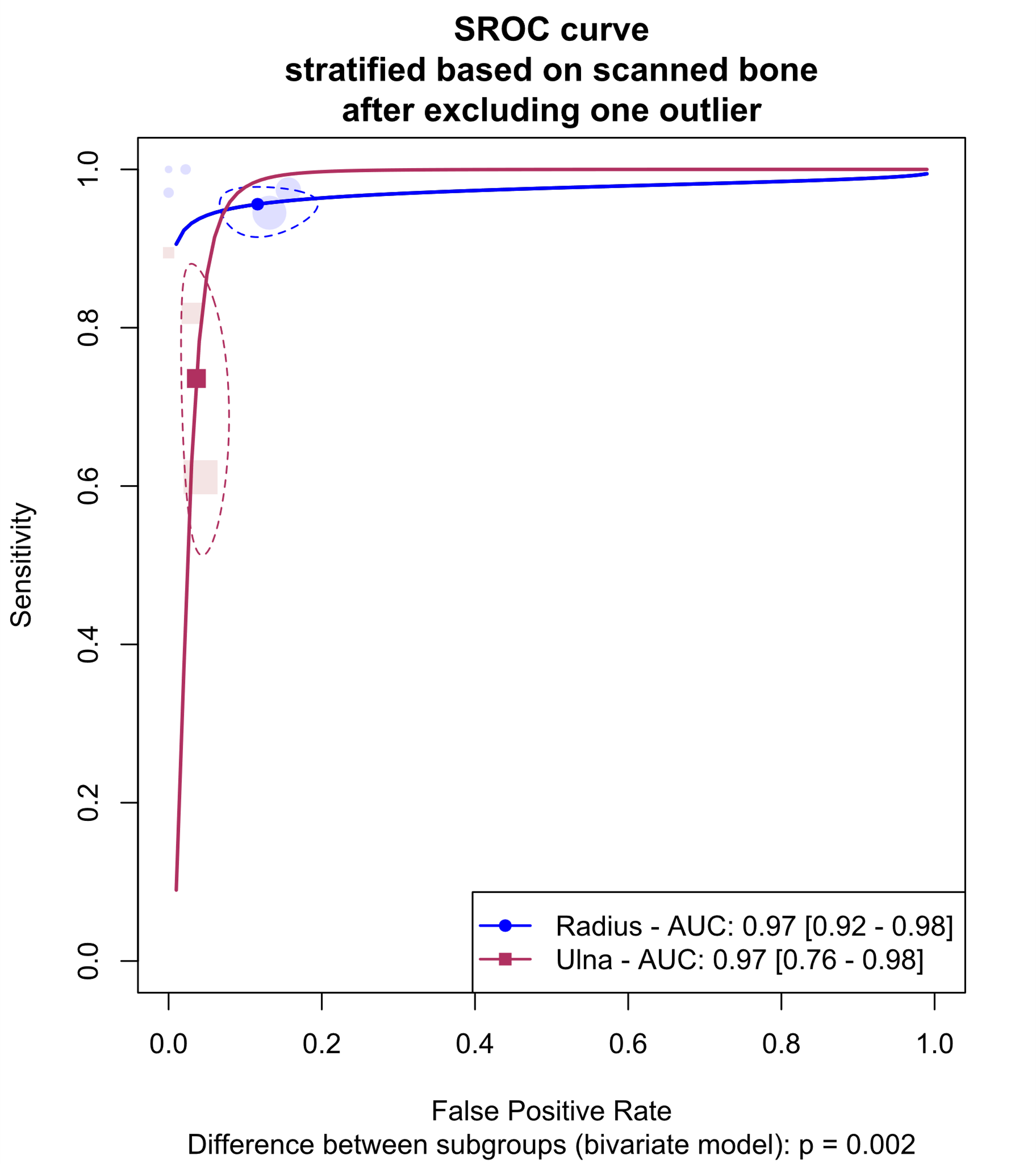


**Supplementary Fig. 13**. Summary receiver operating characteristic curve (SROC) of diagnostic test accuracy (DTA) subgroup meta-analysis comparing the performance of ultrasound to detect fractures of radius and ulna after excluding one outlier study. AUC - Area Under the Curve


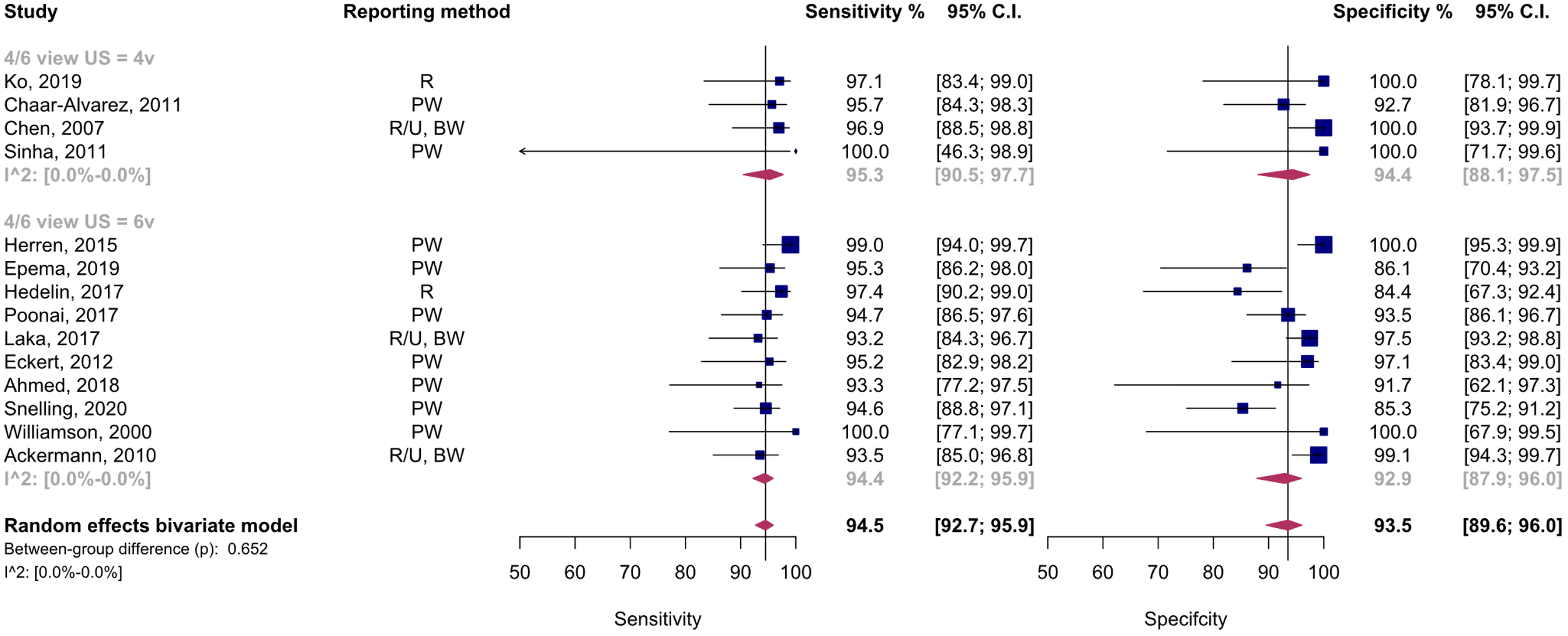
 **Supplementary Fig. 14**. Forest plot and summary statistics of diagnostic test accuracy (DTA) subgroup meta-analysis comparing the performance of 4-view and 6-view US examinations to detect distal forearm fractures. CI - Confidence Interval, R - Radius, U - Ulna, US – Ultrasound, BW - Bone-wise, PW - Patient-wise


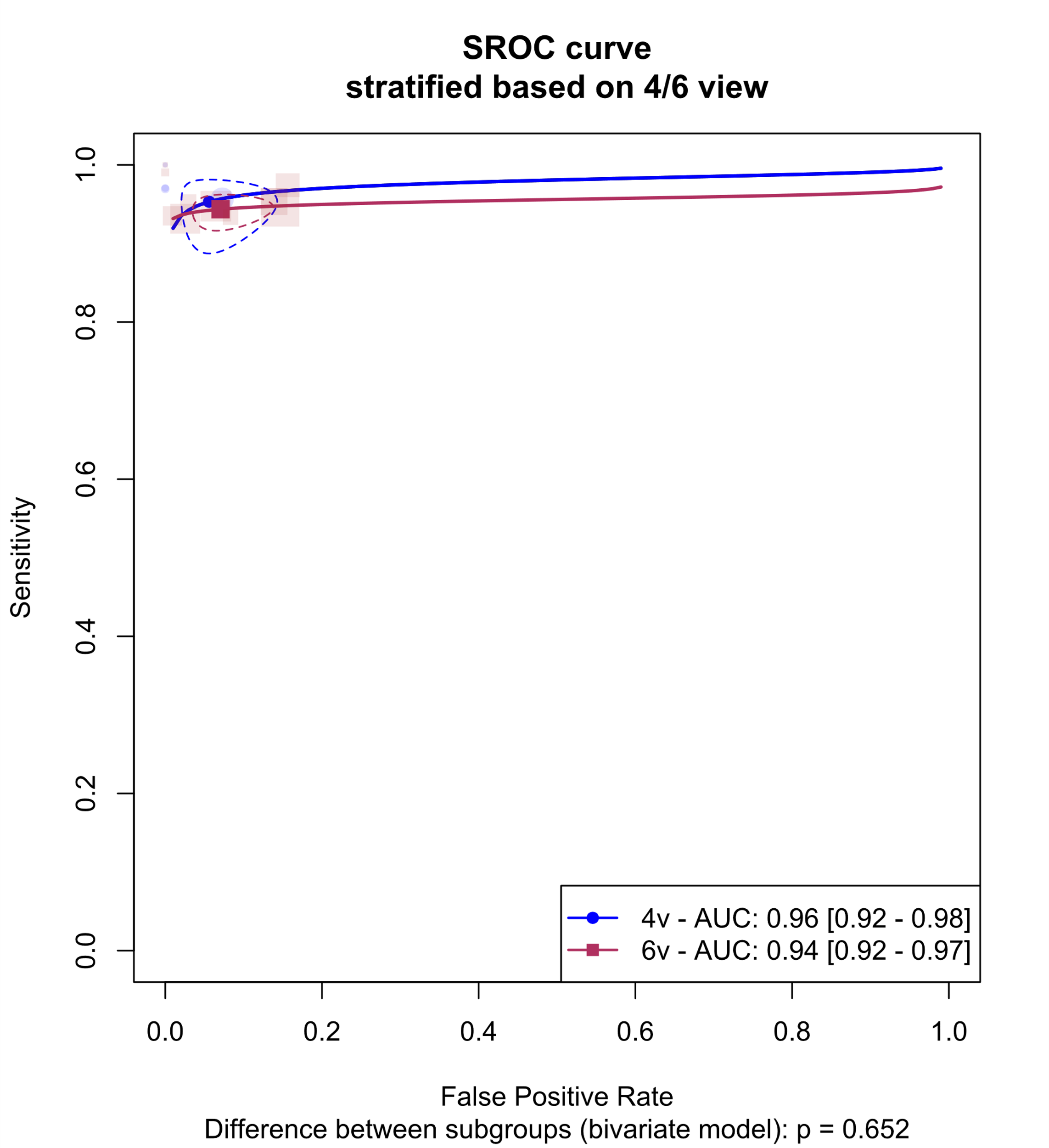
 **Supplementary Fig. 15**. Summary receiver operating characteristic curve (SROC) of diagnostic test accuracy (DTA) subgroup meta-analysis comparing the performance of 4-view and 6-view ultrasound in detecting distal forearm fractures. AUC - Area Under the Curve


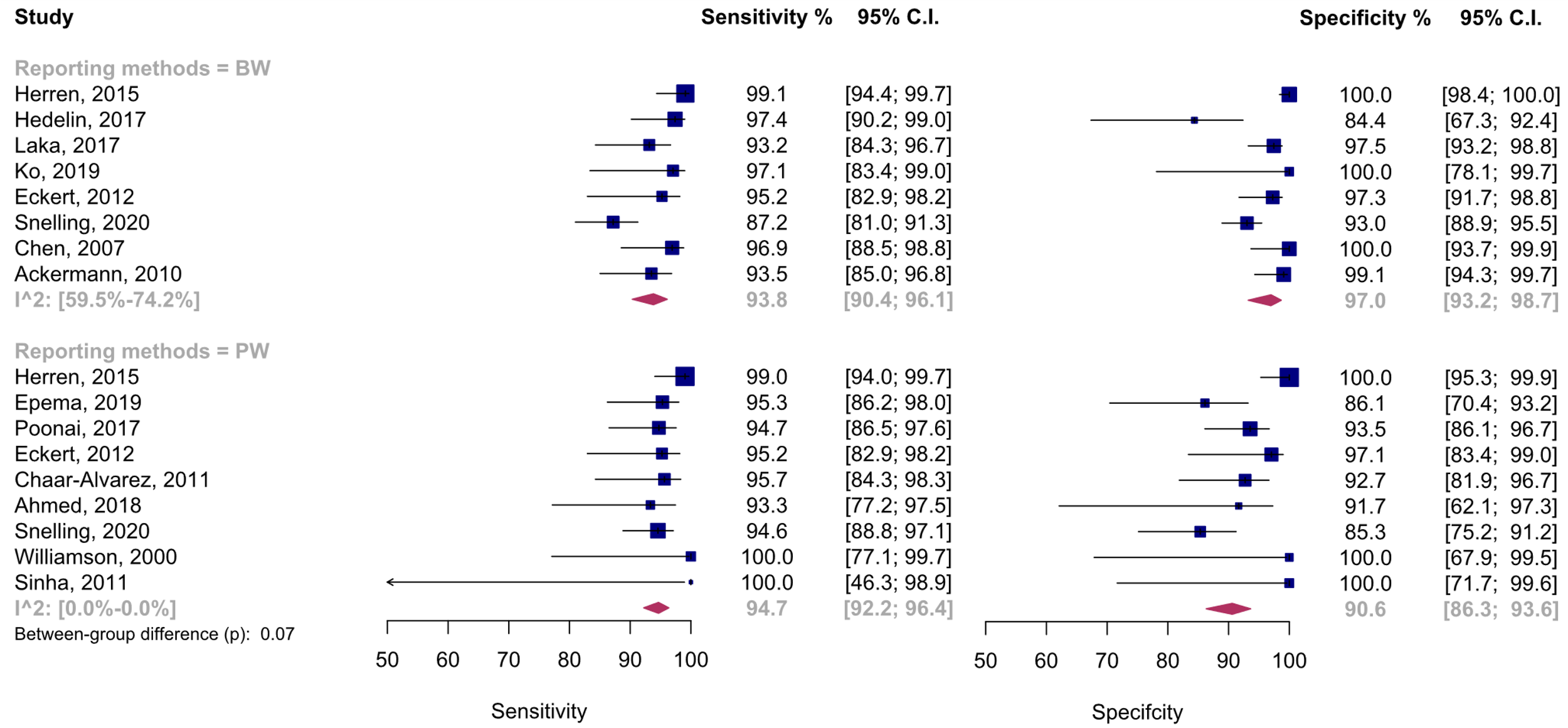
 **Supplementary Fig. 16.** Forest plot and summary statistics of diagnostic test accuracy (DTA) subgroup meta-analysis comparing ultrasound examination results reporting methods - Patient-Wise Versus Bone-Wise. CI - Confidence Interval, R - Radius, U - Ulna, BW - Bone-wise, PW - Patient-wise


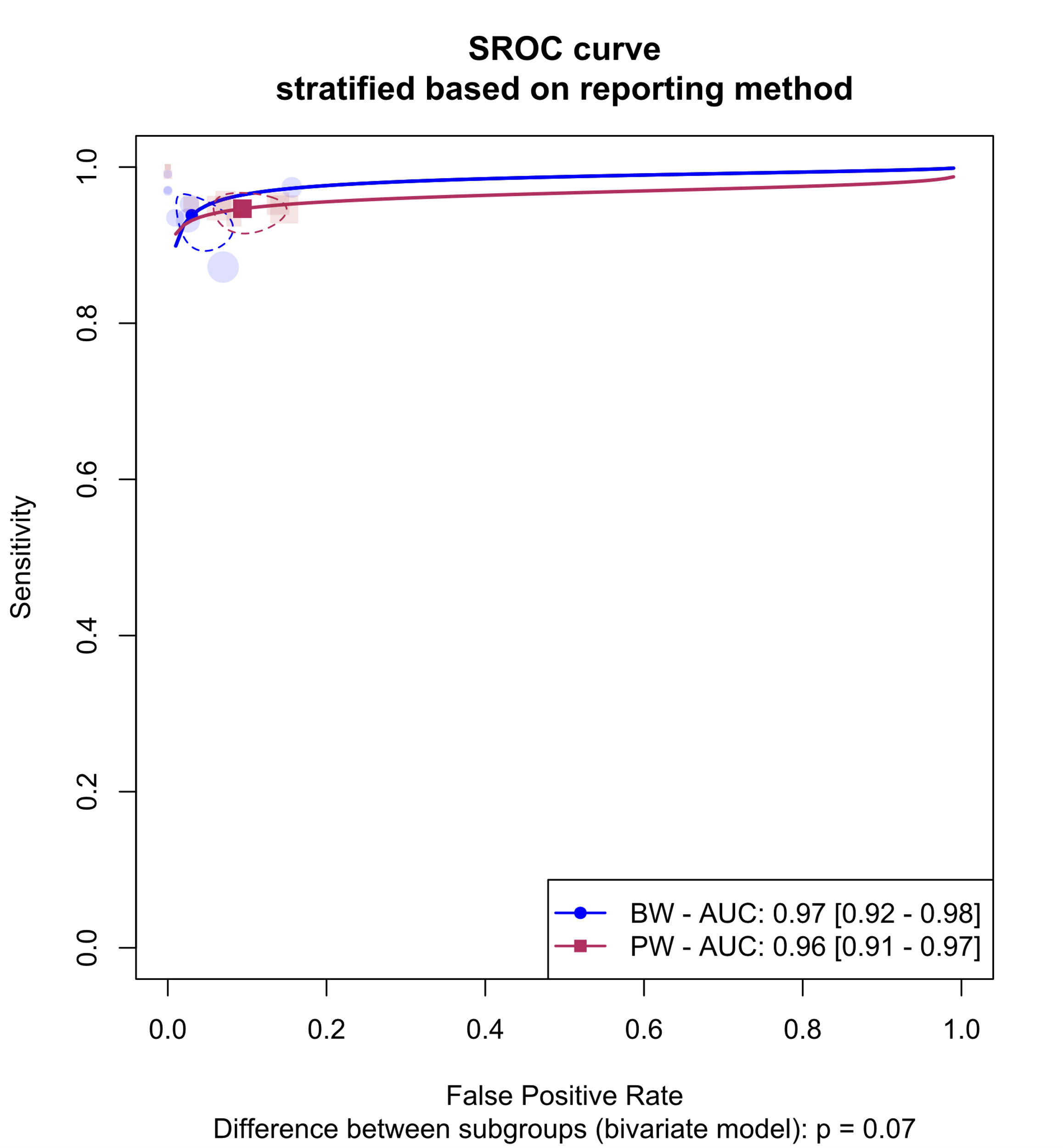
 **Supplementary Fig. 17**. Summary receiver operating characteristic curve (SROC) of diagnostic test accuracy (DTA) subgroup meta-analysis comparing ultrasound examination results reporting methods - Patient-Wise Versus Bone-Wise. AUC - Area Under the Curve, BW - Bone-wise, PW - Patient-wise


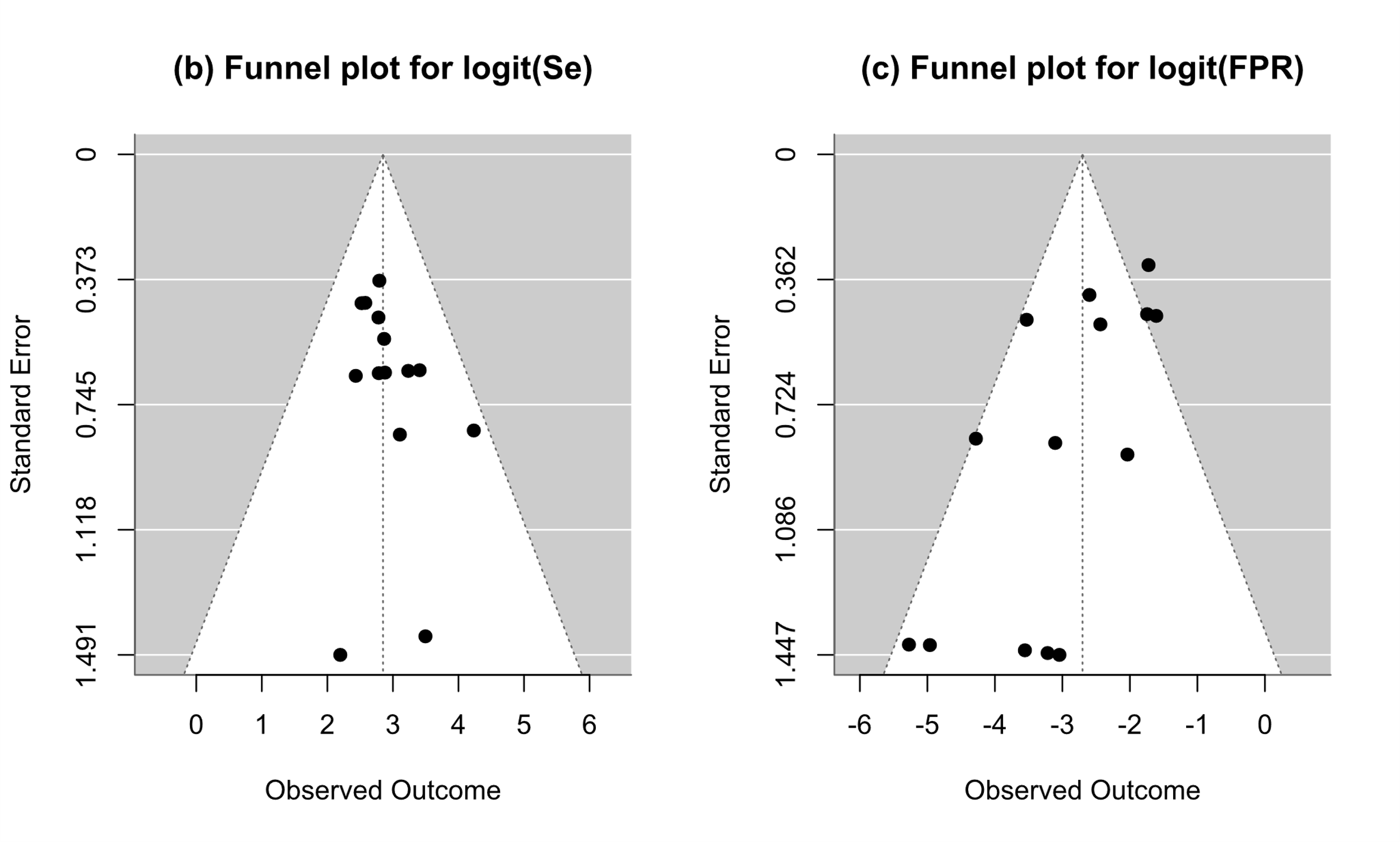


**Supplementary Fig. 18.** Paired funnel plots for assessing potential publication bias/small study effect in the general meta-analysis. Abbreviations: FPR - False Positive Rate, Se – Sensitivity
